# Development of formaldehyde-killed and alum-adjuvanted multicomponent *Salmonella* Enteritidisphage-type vaccines in chickens

**DOI:** 10.5455/javar.2025.l989

**Published:** 2025-12-25

**Authors:** Amanullah Akhtar, Mohd Hair-Bejo, Elawad A. Hussein, Siti Khairani Bejo, Zunita Zakaria

**Affiliations:** 1 Department of Veterinary Pathology and Microbiology, Faculty of Veterinary Medicine, Universiti Putra Malaysia, Serdang, Malaysia; 2 Department of Veterinary Pathology, Gomal College of Veterinary Sciences, Gomal University, Dera Ismail Khan, Pakistan; 3 Department of Veterinary Pathology and Microbiology, Institute of Bioscience, Universiti Putra Malaysia, Serdang, Malaysia

**Keywords:** *Salmonella* Enteritidis(SE), phage types (PTs), chick, killed, alum adjuvant

## Abstract

**Objective:**

The study objectives were to develop multicomponent formaldehyde-killed and alum-adjuvanted *Salmonella* Enteritidis(SE) phage-type vaccines in chickens.

**Materials and Methods:**

SEPTs 35, 7, 6A, 3A, and 1 were killed, mixed to prepare six combinations, namely, V635, V671, V673, V675, and V613 and administered (0.20 ml) to chicks (*n* = 124) of 6 groups (CV635, CV671, CV673, CV675, and CV613), and one non-administered group as a control. Other chicks underwent 0.20 ml with SE 6A per chick (1,010 cfu per ml) as a challenge and were labeled CVZC, CV635C, CV671C, CV673C, CV675C, and CV613C. The blood, swab of cloaca, liver, spleen, digesta of the middle part of the intestine, and digesta of the cecum samples were collected for *Salmonella* detection. The caecum, bursa of Fabricius, liver, spleen, and ileum tissues were collected for histopathological examination.

**Results:**

*Salmonella* was detected (100%) from the digesta of the middle part of the intestine, swabs of the cloaca, digesta of the caecum, and blood, spleen, and liver samples in the CVZC. *Salmonella* was not detected from the 3 (50%), 2 (33%), and 1 (17%) samples in the CV671C and CV673C, CV613C and CV635C, and CV675C, respectively. Histopathological changes were mild (lesion scoring of 0.4/3.0) and recorded in the group CVZC in the ileum, cecum, and bursa of Fabricius on days 7 and 14 pc, respectively.

**Conclusions:**

All combinations of killed SEPTs could protect the chick against SE infection. However, V673 and V671 products are safer and more effective compared to other products.

## Introduction

*Salmonellosis*, a zoonotic disease, is the most prevalent foodborne disease worldwide [1]. This microbe is ubiquitous, comprising more than 2,600 typhoidal and non-typhoidal serovars [2]. In general, diseases caused by *Salmonella* spp. resulted in one of the largest economic burdens, according to records from the U.S. Department of Agriculture [3].

The high prevalence of *Salmonella* infection, accompanied at times by mortality cases, in addition to the increased emergence of multidrug-resistant strains, was the primary factor driving the development of vaccines against *Salmonella* infection [1]. Many types of vaccines have been successfully developed, including live-attenuated vaccines. In this type, the vaccine prompts both cellular and humoral immunity by exposing the antigen to class I and class II MHC molecules [4]. Additionally, killed vaccines were successfully developed by growing the microbes, then killing them with chemicals or heat, and administering them to stimulate the production of antibodies [5]. Nevertheless, the available systemic vaccines have significant disadvantages, including reduced effectiveness in young individuals and a lack of cross-protection among different strains [6].

According to the fact that vaccines based on multiple antigens often induce a higher immune response than that triggered by every single component. This is due to the cooperative and synergistic actions of the antibodies in tackling the infection [7]. It was expected that the development of a multicomponent vaccine composed of a combination of different phage types (PTs) against *Salmonella* Enteritidis(SE)isolated in Malaysia would be effective. In other words, a multicomponent vaccine might overcome the disadvantage of the reduced effectiveness of the previous vaccines. The objectives of this study were to develop formalin-killed and alum-adjuvanted multicomponent vaccines against SE infection in chickens.

## Materials and Methods

### Ethical approval

The procedures of the experiment in this study strictly follow the Universiti Putra Malaysia’s Research Policy on Animal Ethics, under permit number RPAE1370.

### Propagation and inactivation of SEPTs

All SEPTs—PT 6A (UPM-0527), PT 7 (UPM-0530),PT 35 (UPM-0525), PT 3A (UPM-0541), and PT 1 (UPM-05)—were isolated in Malaysia in 2005. Next, the samples were sent to Britain for identification at the Enteric Pathogens Laboratory, Infection Department, Colindale Avenue in London [8]. All the SEPTswere propagated, identified, confirmed, and killed in formaldehyde (0.7%) as previously described [8].

### Preparation of combinations of killed SEPTs

Four liters of the harvest from three different killed SEPTs, 6A, 7, and 1, were mixed in a plastic drum to make a combination designated as V671, which represented formalin-killed SEPTs 6A, 7, and 1. Similarly, a mixture of killed SEPTs 6A, 3A, and 35 was prepared and designated as V635; killed SEPTs 6A, 7, and 3A were designated as V673; killed SEPTs 6A, 7, and 35 were designated as V675, and killed SEPTs 6A, 1, and 3A were designated as V613. An adjuvant solution of 10% aluminum potassium sulfate (Alum) was prepared and mixed with the killed SEPTs as previously described [8].The products were named according to V671, V635, V673, V675, and V613.

### Designof trial

One hundred twenty-four 1-day-old specific pathogen-free (SPF) chicks were used in the study. To determine the SPF status of the chicks, four chicks were sacrificed at the beginning of the trial. The mass, blood, and cloacal swab samples were taken. Samples for *Salmonella* isolation and histopathological examination were also taken. The other chicks were then grouped into six groups of 20 chicks each, namely CV635, CV671, CV673, CV675, CV613, and CVZ, representing the chicks administered V635, V671, V673, V675, and V613 products, and the control or non-administered chicks, respectively.

The chicks were administered accordingly to the killed SEPTs with a dosage of 0.10 ml per chick (10_10_ cfu per ml) subcutaneously on the neck region. Different groups were kept in separate cages. Water and antibiotic-free feed were given *ad libitum* during the trial. On 14 days post-administration (pa), sampling was conducted prior to pathogenic SEPTs 6A (UPM-0527). The mass, blood, and cloacal swab samples of four chicks from each group were collected prior to sacrifice. The chicks were sacrificed by cervical dislocation, and a necropsy was conducted. Gross lesions were recorded, and samples were taken for *Salmonella* detection, histopathological examination, and scoring of lesions. The serum samples were stored at −20°C prior to the detection of *Salmonella* antibodies using the ELISA technique.

Sixteen chicks remained in every group prior to undergoing pathogenic SE on 14 days pa. The chicks in every group were divided into two groups, namely the pathogenic SE PT and the groups without pathogenic SE PT of eight chicks from every group, and kept in different rooms. The chicks orally underwent pathogenic doses of 0.20 ml per chick (109 cfu per ml) ofSEPTs 6A (UPM-0527) and were identified as CV635C, CV671C, CV673C, CV675C, CV613C, and CVZC. Such groups were administered with V635, V671, V673, V675, and V613, respectively, and underwent pathogenic doses of SEPTs 6A (UPM-0527) as a challenge. The CVZC is the control or non-administered group, but underwent the pathogenic dose of SEPTs 6A. The chicks were monitored for any unusual clinical signs during the post-pathogenic SE PT6A (pp) period. Four chicks from both the pathogenic SE PT6A groups and the groups without pathogenic SE PT6A were sacrificed at 7 and 14 days post-hatch for sampling. The mass, blood, and cloacal swab samples were taken prior to sacrifice by cervical dislocation. On necropsy, the gross lesions were recorded, and samples were taken for *Salmonella* isolation, histopathological examination, and scoring of lesions (Table 1).

**Table 1.** Design of the trial.

Groups	Different products of killed SE PTs	Administration with killed SE PTs or undergoing pathogenic SE PT 6A	Chicks’ numbers sacrificed on certain days postadministration			
			D_0_	D_14_	D_21_	D_28_
CV635	Killed SE PT 6A, 3A and 35	Administered with killed SE PT but no pathogenic dose of SE PT 6A	0	4	4	4
CV635C	killed SE PT 6A, 3A and 35	administered with killed SE PT then pathogenic dose of SE PT 6A	0	0	4	4
CV671	killed SE PT 6A, 7 and 1	administered with killed SE PT but no pathogenic dose of SE PT 6A	0	4	4	4
CV671C	killed SE PT 6A, 7 and 1	administered with killed SE PT then pathogenic dose of SE PT 6A	0	0	4	4
CV673	killed SE PT 6A, 7 and 3A	administered with killed SE PT but no pathogenic dose of SE PT 6A	0	4	4	4
CV673C	killed SE PT 6A, 7 and 3A	administered with killed SE PT then pathogenic dose of SE PT 6A	0	0	4	4
CV675	killed SE PT 6A, 7 and 35	administered with killed SE PT but no pathogenic dose of SE PT 6A	0	4	4	4
CV675C	killed SE PT6A, 7 and 35	administered with killed SE PT then pathogenic dose of SE PT 6A	0	0	4	4
CV613	killed SE PT 6A, 1 and 3A	administered with killed SE PT but no pathogenic dose of SE PT 6A	0	4	4	4
CV613C	killed SE PT 6A, 1 and 3A	administered with killed SE PT then pathogenic dose of SE PT 6A	0	0	4	4
CVZ	Control	administered with killed SE PT but no pathogenic dose of SE PT 6A	4	4	4	4
CVZC	Control	Non- administered with killed SE PT and no pathogenic dose of SE PT 6A	0	0	4	4

Killed SEPTs were administered to 1-day-old chicks, and then the chicks underwent pathogenic SEPT 6A on 14 days post-administration. D_0_, D_14_, D_21_ and D_28_ represent days 0, 14, 21, and 28, respectively.

### Isolation and identification

The blood, swab of cloaca, liver, spleen, digesta of the middle part of the intestine, and digesta of the cecum samples were collected for the *Salmonella* isolation and identification as previously described [9].

### Histopathological examination and scoring of lesions

The liver tissues, spleen tissues, bursa of Fabricius tissues, ileum tissues, and caecum tissues were fixed in 10% buffered formaldehyde, embedded in paraffin wax, and stained with hematoxylin and eosin for histopathological examination and scoring of lesions: normal tissue (0), mild change (1), moderate change (2), and severe change (3). (a) Liver: lesions were scored as 0 = normal tissue or no histopathological changes observed; 1 = mild congestion, mild degeneration, mild cellular infiltration, and mild necrosis; 2 = moderate congestion or hemorrhage, moderate degeneration, fatty changes, mild to moderate cellular infiltration, and moderate necrosis; 3 = severe congestion or hemorrhage, moderate to numerous cellular infiltration, and severe necrosis. (b) Spleen: lesions were scored as 0 = normal tissue or no histopathological changes observed; 1 = mild congestion, mild degeneration, mild cellular infiltration, and mild necrosis; 2 = moderate congestion or hemorrhage, moderate degeneration, mild to moderate cellular infiltration, and mild to moderate necrosis; 3 = extensive congestion or hemorrhage and moderate to numerous cellular infiltrations and extensive necrosis. (c) Bursa of Fabricius:lesions were scored as 0 = normal tissue or no histopathological changes observed; 1 = mild congestion, mild degeneration, necrosis, and mild cellular infiltration; 2 = moderate congestion or hemorrhage, moderate degeneration, moderate cellular infiltration, and necrosis; 3 = severe congestion or hemorrhage, moderate to severe cellular infiltration, and depletion. (d) Ileum: lesions were scored as 0 = normal tissue or no histopathological changes observed; 1 = mild congestion, mild degeneration, mild cellular infiltration, and mild necrosis; 2 = moderate congestion or hemorrhage, moderate degeneration, mild to moderate cellular infiltration, reactive goblet cells, sloughing of enterocytes, and moderate necrosis; 3 = severe congestion or hemorrhage and moderate to severe cellular infiltration, severe active goblet cells, and severe necrosis associated with sloughing of epithelial cells. (e) Caecum: lesions were scored as 0 = normal tissue or no histopathological changes observed; 1 = mild congestion, mild degeneration, mild cellular infiltration, and mild necrosis; 2 = moderate congestion or hemorrhage, moderate degeneration and necrosis, mild to moderate cellular infiltration, reactive goblet cells, and sloughing of villi; 3 = severe congestion or hemorrhage and mild to moderate cellular infiltration, severe reactive goblet cells, severe sloughing of villi, and necrosis.

### Detection of SE antibody

The serum samples were detected for SE antibodyusing the Biocheck ELISA technique as described previously [10].

### Turkey’s honest significant difference pairwise multiple procedure

The data were statistically analyzed with Tukey’s honest significant difference pairwise multiple comparison procedure.

## Results

### Clinical signs and mortality cases

Groups without pathogenic SE PT6A dose: CVZ, CV635, CV671, CV673, CV675, and CV613 showed no unusual mortality cases during the trial.

Group with pathogenic SE PT6A:In the CVZC group, starting from 2 days pp, the chicks were depressed, anorexic, and had diarrhea, but this was not recorded after 7 days of the trial. On 3 days pp, the chicks showed ruffled feathers. No mortality was observed during the trial. However, in CV635C, CV675C, and CV613C, only signs of depression and anorexia were recorded at 1 and 2 days pp, and CV671C only at 1 day pp. In CV675C, ruffled feathers were recorded after 3 days pp. Mortality cases were not observed during the trial.

### Mass

**Figure 1.**
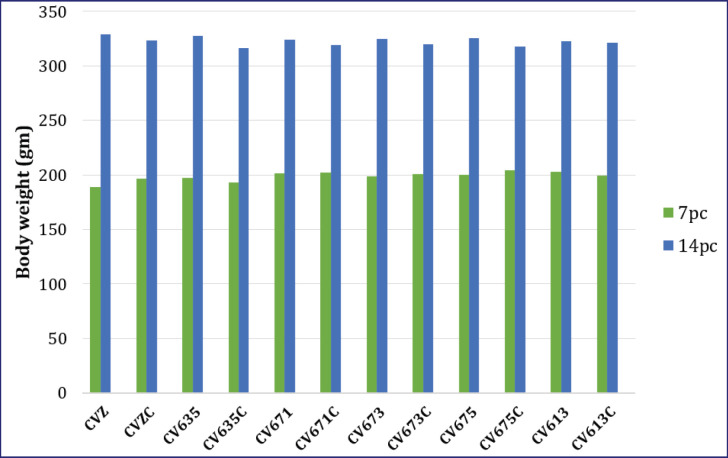
Mass (body weight) of chickens (mean ± SEM in gm) from different groups after administration of killed SE PTs, then pathogenic SE PT6A or those without pathogenic SE PT6A on 7- and 14-day pp.

The mass in CVZ was increased continuously during the trial. It was 37.50 ± 2.10 gm and 129.00 ± 4.80 gm on 0 and 14 days pa, respectively, and 189.00 ± 9.60 gm and 329.00 ± 6.70 gm on 7 and 14 days pp, respectively. In the group CVZC, the mass was 196.30 ± 8.30 gm and 323.00 ± 2.3 gm on 7 and 14 days pp, respectively. It was not significantly different (*p* < 0.05) in comparison with the CVZ on 7- and 14-day pp (Fig. 1).

The mass in CV635 was 37.50 ± 2.10 gm and 127.80 ± 1.00 gm on 0 and 14 days pp, respectively, and 197.30 ± 5.50 gm and 327.50 ± 4.70 gm on 7 and 14 days pp, respectively. In the CV635C, the mass was 193.30 ± 3.90 gm and 316.30 ± 5.80 gm on 7 and 14 days pp, respectively. It was not significantly (*p* < 0.05) different in comparison with CV635 (Fig. 1).

The mass in CV671 was 37.50 ± 2.10 gm and 128.00 ± 1.80 gm on 0 and 14 days pa, respectively, and 201.30 ± 2.30 gm and 324.00 ± 3.60 gm on 7 and 14 days pp, respectively. In the CV671C, the mass was 202.00 ± 3.70 gm and 319.30 ± 5.80 gm on 7 and 14 days pp, respectively. It was not significantly (*p* < 0.05) different in comparison with CV671 (Fig. 1).

The mass in CV673 was 37.50 ± 2.1 gm and 126.00 ± 2.50 gm on 0 and 14 days pa, respectively, and 198.50 ± 4.40 gm and 325.00 ± 12.40 gm on 7 and 14 days pp, respectively. The mass of CV673C was 201.00 ± 2.90 gm and 320.00 ± 9.10 gm on 7 and 14 days pp, respectively. It was not significantly (*p* < 0.05) different in comparison with CV673 (Fig. 1).

The mass in CV675 was 37.50 ± 2.10 gm and 127.00 ± 4.40 gm on 0 and 14 days pa, respectively, and 200.00 ± 5.00 gm and 325.30 ± 14.80 gm on 7 and 14 days pp, respectively. The mass in CV675C was 204.50 ± 8.80 gm and 318.80 ± 7.90 gm on 7 and 14 days pp, respectively. It was not significantly (*p* < 0.05) different in comparison with CV675 (Fig. 1).

The mass in CV613 was 37.50 ± 2.10 gm and 127.30 ± 2.70 gm on 0 and 14 days pa, respectively, and 202.50 ± 3.40 gm and 322.80 ± 7.5 gm on 7 and 14 days pp, respectively. The mass in CV613C was 199.00 ± 6.40 gm and 321.00 ± 7.90 gm on 7 and 14 days pp, respectively. It was not significantly different (*p* < 0.05) in comparison with CV613 (Fig. 1).

### Detection of Salmonella

**Figure 2.**
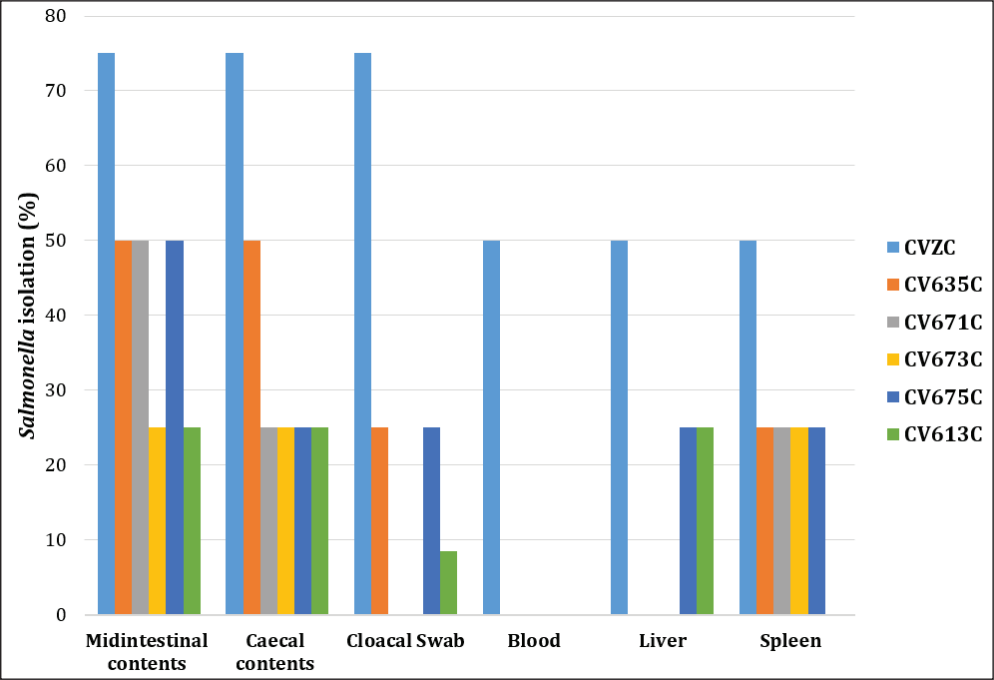
*Salmonella* isolated from different tissues collected from chickens on 7 days pp after administration of killed SE PTs and then Pathogenic SE PT6A or those who did not undergo pathogenic SE PT6A. Midintestinal contents represent digesta of the middle part of the intestine. Cecal contents represent digesta of the cecum.

**Figure 3.**
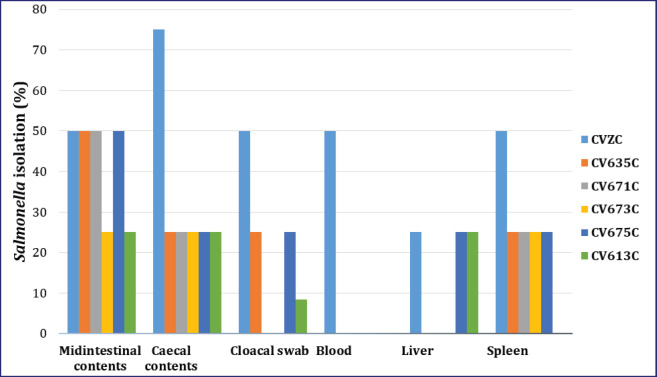
*Salmonella* detected in different tissues collected from chickens on 14 days pp after administration of killed SE PTs or pathogenic SE PT6A or those that did not undergo pathogenic SE PT6A. Midintestinal contents represent digesta of the middle part of the intestine. Cecal contents represent digesta of the cecum.

The microbe was not detected at all in the samples collected throughout the trial in the CVZ. It was also not detected at all in the tissues before administration of SE on 0 day pa. In contrast, *Salmonella* was detected in all samples collected in the CVZC (Tables 2, 3 and Figs. 2, 3). *Salmonella* was detected 75% and 50% on 7 and 14 days pp, respectively, from swabs of the cloaca and middle part of the intestine digesta, respectively. *Salmonella* was detected 75% on days 7 and 14 pp from digesta of the caecum. *Salmonella* was detected 50% on 7 and 14 days pp from blood and spleen samples. Eventually *Salmonella* was detected in 50% and 25% on 7 and 14 days pp, respectively, from liver samples.

**Table 2.** *Salmonella* detected in different tissues of chickens on 7 days pp after administration with killed SE PTs and then pathogenic SE PT6A.

Organs/ samples	Groups of chickens that underwent pathogenic SE PT6A then *Salmonella* was isolated					
	CVZC	CV635C	CV671C	CV673C	CV675C	CV613C
Digesta of middle part of intestine	+ve	+ve	+ve	+ve	+ve	+ve
Digesta of caecum	+ve	+ve	+ve	+ve	+ve	+ve
Swab of cloaca	+ve	+ve	−ve	−ve	+ve	+ve
Blood	+ve	−ve	−ve	−ve	−ve	−ve
Liver	+ve	−ve	−ve	−ve	+ve	+ve
Spleen	+ve	+ve	+ve	+ve	+ve	−ve
Number of positives (+ve)	6	4	3	3	5	4

**Table 3:** :*Salmonella* detection in different tissues of chickens on 14 days pp after administration of killed SE PTs then pathogenic SE PT6A.

Organs/ samples	Groups of chickens with killed SE PTs and pathogenic SE PT6A then *Salmonella* was isolated					
	CVZC	CV635C	CV671C	CV673C	CV675C	CV613C
Digesta of middle part of intestine	+ve	+ve	+ve	+ve	+ve	+ve
Digesta of caecum	+ve	+ve	+ve	+ve	+ve	+ve
Swab of cloaca	+ve	+ve	−ve	−ve	+ve	+ve
Blood	+ve	−ve	−ve	−ve	−ve	−ve
Liver	+ve	−ve	−ve	−ve	+ve	+ve
Spleen	+ve	+ve	+ve	+ve	+ve	−ve
Number of positives (+ve)	5	4	3	3	5	4

The microbe was not detected in any of the samples collected during the trial in CV635. However, *Salmonella* was detected in some samples with different percentages in the CV635C. It was detected at 50% on 7 and 14 days in 7 pp from the middle part of the intestine digesta samples. It was detected 50% and 25% on 7 and 14 days pp, respectively, from the digesta of cecum samples. It was detected at 25% on 7 and 14 days pp from spleen and swabs of cloaca samples. However, it was not detected in blood and liver samples on 7- and 14-day pp (Tables 2, 3 and Figs. 2, 3).

The microbe was not detected in any of the samples collected during the trial in the CV671. It was detected at 50% on 7 and 14 days pp from digesta of the middle part of intestine samples in the CV671C. It was detected 25% in 7- and 14 days pp from the spleen and the digesta of the cecum samples. However, it was not detected from blood, liver, and swabs of cloaca samples on 7 and 14 days pp (Tables 2, 3 and Figs. 2, 3).

The microbe was not detected in any of the samples collected during the trial in the CV673. It was detected at 25% on 7 and 14 days pp from the spleen, digesta of the middle part of the intestine, and digesta of caecum samples in the CV673C. While it was not detected on 7 and 14 days pp from blood, liver, and swabs of cloaca samples (Tables 2, 3 and Figs. 2, 3).

The microbe was not detected in all samples collected during the trial in the CV675. It was detected at 50% on 7 and 14 days pp from the digesta of the middle part of the intestine samples in the CV675C. Moreover, it was detected 25% in 7 and 14 days pp from the liver, spleen, swabs of cloaca, and digesta of caecum samples. However, it was not detected on days 7 and 14 in 7 pp from blood samples (Tables 2, 3 and Figs. 2, 3).

The microbe was not detected in any of the samples collected during the trial in CV613. It was detected at 25% on 7 and 14 days pp from the liver, digesta of the middle part of the intestine, and digesta of caecum samples in the CV613C. It was not detected on 7 and 14 days pp from blood, spleen, and swabs of cloaca samples (Tables 2, 3 and Figs. 2, 3).

Based on the results of *Salmonella* isolated from different samples on 7- and 14-day pp after administration of chickens with killed SE PTs and pathogenic SE PT6A, it was demonstrated that the CV673 and CV671 products were the best for protection of the chickens against the microbe (Tables 2, 3).

### Gross lesions

Gross lesions were not recorded in all groups of chickens during the trial.

### Histopathological lesions

#### Ileum

**Figure 4.**
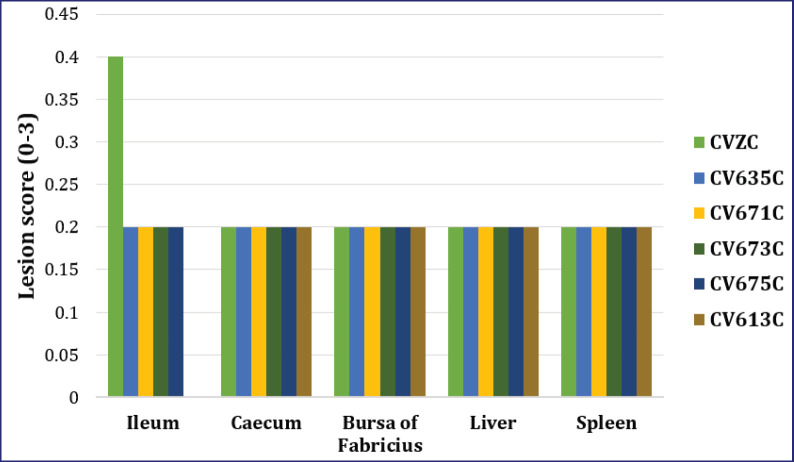
Lesion scoring of different organs from different groups of chickens on 7 days pp after administration of killed SE PTs and pathogenic SE PT6A.

**Figure 5.**
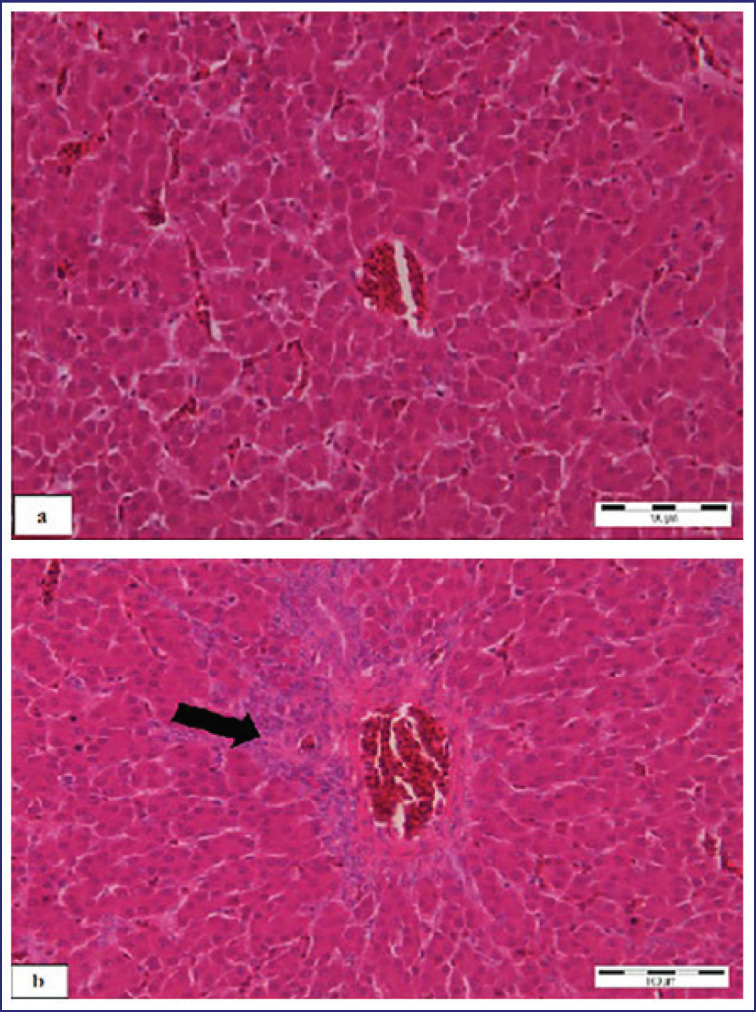
Lesion scoring of different organs collected from different groups of chickens on 14 days pp after administration of killed SE PTs and pathogenic SE PT 6A.

The histopathological lesions were not detected during the trial; consequently, the scoring was 00 ± 00 in the CVZ. However, the score was 0.40 ± 0.20 and 0.20 ± 0.20 on 7 and 14 days pp, respectively, in the CVZC (Figs. 4, 5).In all groups of chicks administered with different killed SE PTs without pathogenic SE PT6A (CV635, CV671, CV673, CV675, and CV613), no lesions were detected (scoring of 00 ± 00) during the trial.However,in the pathogenic SE PT6A groups, lesion scores of 0.20 ± 0.20 were recorded on 7 and 14 days pp, respectively, in the CV635C and CV675C. The scores were recorded as 0.20 ± 0.20 and 0.00 ± 0.00 on 7- and 14-day pp, respectively, in the CV671C, CV673C, and CV613C (Figs. 4, 5).

#### Cecum

The microscopic changes were not detected during the trial; thus, the scoring was 00 ± 00 in the CVZ. However, scoring was 0.20 ± 0.20 and 0.40 ± 0.20 on 7 and 14 days pp, respectively, in the CVZC (Figs. 4, 5). In all groups of chicks with different killed SE PTs without pathogenic SE PT6A (CV635, CV671, CV673, CV675, and CV613), no lesions were detected (scoring of 00 ± 00) during the trial. However,in the CV635C, CV675, and CV613C, lesion scoring of 0.20 ± 0.20 was recorded on 7- and 14-day pp. In CV671C and CV673C, the score was 0.20 ± 0.20 and 0.00 ± 0.00 on 7- and 14-day pp, respectively (Figs. 4, 5).

#### Bursa of Fabricius

The lesions were not detected during the trial, and so they were scored 00 ± 00 in the CVZ. However, the scoring was 0.20 ± 0.20 and 0.40 ± 0.20 on 7 and 14 days pp, respectively, in the CVZC (Figs. 4, 5). The lesions were also not recorded. Hence, the scoring was 00 ± 00 in the CV635, CV671, CV673, CV675, and CV613 during the trial. Eventually, the scoring was 0.20 ± 0.20 on 7 and 14 days pp in the CV635C, CV671C, CV673C, CV675C, and CV613C (Figs. 4, 5).

#### Liver

**Figure 6.**
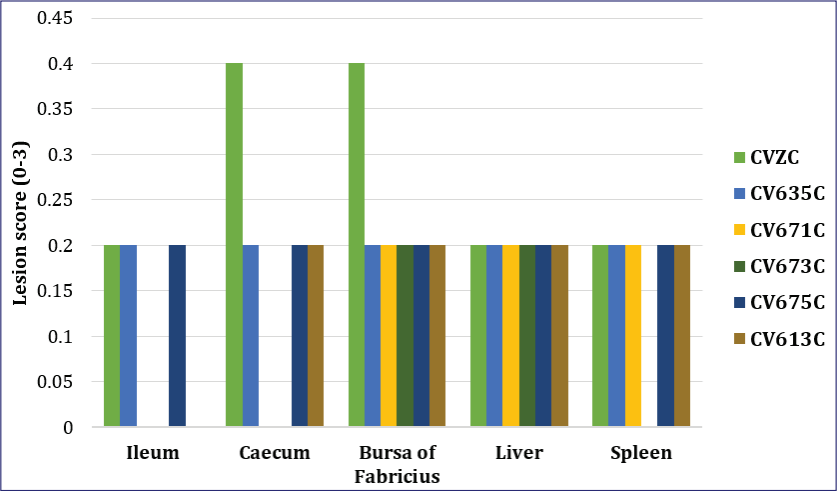
(a) Liver of chicks inoculated with different inactivated products without challenge (group CV673), microscopic lesions were not detected (b) Liver of chicks inoculated with different inactivated products and challenge (group CV673C). Moderate heterophilic infiltration (black arrow). Scale bar = 100 μm. HE staining.

The lesions were not detected during the trial, and the scoring was 00 ± 00 in the CVZ. Lesion scoring of 0.20 ± 0.20 was recorded on 7 and 14 days pp, respectively, in the CVZC (Figs. 4, 6). No lesions were detected (scoring of 00 ± 00) in the CV635, CV671, CV673, CV675, and CV613 during the trial. Lesion scoring of 0.20 ± 0.20 was recorded on 7 and 14 days pp in the CV635C, CV671C, CV673C, CV675C, and CV613C (Figs. 4, 6).

#### Spleen

The microscopic changes were not detected during the trial, viz., it was scored 00 ± 00 in the CVZ. Lesion scoring of 0.20 ± 0.20 was recorded on 7 and 14 days pp. Lesion scoring was not detected (scoring of 00 ± 00) in CV635, CV671, CV673, CV675, and CV613 during the trial. Lesion scoring of 0.20 ± 0.20 was recorded on 7 and 14 days pp in all groups, except it was not detected in the CV673C on 14 days pp (Figs. 4, 5).

### Detection of SE antibody

SE antibody was not detected in any of the chicken groups during the trial.

## Discussion

The study demonstrated that the combinations of killed SEPT isolates were safe and effective to reduce the clinical signs, lesions, and detection of *Salmonella* following the pathogenic dose of SEPT 6A on 14 days pa. All the combinations had the ability to mitigate the clinical signs, while the combination of V673 had completely protected SPF chicks from the clinical disease. Consequently, V673 might help to control SE infection in poultry and foodborne *Salmonellosis*. Since different SEPTs vary in their virulence [11] and hence in their immunogenicities [12], the variations among different combinations of killed SEPTs in this study might be due to some differences in their immunogenicities [13].

In a previous study in Brazil, it was concluded that three different killed SE vaccines could decrease the presence of SE in both the chicks and the eggs. Nevertheless, the author recommended general hygiene and disinfection practices beside vaccination for better results. This was because a very small amount of *Salmonella* was isolated in the spleen, liver, ovary, and caeca of the birds [14]. In another study, it was concluded that a killed trivalent *Salmonella enterica* gave a vaccine for protection with considered safety and efficacy against the colonization of the microbe in the intestine and its attack in the tissues. Thus, it could significantly contribute to the reduction of cases of human food poisoning, in addition to the reduction of antibiotic consumption throughout the productivity age [15].

The mode of action of killed vaccines was to induce an increase in the proportion of circulating monocytes, in addition to prompting a reduction in the percentage of several leukocyte subsets. Eventually, specific serum IgY and mucosal IgA production were also induced [16]. However, higher titers were stimulated in chicks vaccinated with killed vaccines within a shorter time than chicks vaccinated with live vaccines [17].

Mortality cases were not observed in all groups. Therefore, it was difficult to link the mortality control with the administration of bacterins. Nevertheless, it could be due to bacterins or age, because Doan et al. [18] found that bacterins alone or in combination with a live vaccine were effective in preventing mortality induced by infection. Additionally, uniform mass is one important parameter in pullets/layers to achieve peak egg production. It is drastically desired in broilers for profitable farming [19]. In the present study, significant variations in the masses between different groups were not recorded. It was reported previously by Muniz et al. [20] that significant variations were recorded in the mass gains between chicks vaccinated (live non-virulent AWC 591 *Salmonella,* Poulvac^®^ ST, Zoetis, Madison, NJ) and challenged and non-vaccinated chicks and challenged.

The clinical and/or subclinical colonization of SEis a serious concern, as carrier birds can contaminate the environment, and poultry products may lead to foodborne human infections. If a control strategy could eliminate SE from the host, it would be ideal. However, thorough SE control programs have been found to be successful in reducing infections of this microbe in both egg-layers and humans [21].

Different combinations of killed SEPTs could reduce the organ’s colonization, fecal shedding, systemic spread, and egg contamination [22]. Thus, it could be useful to reduce the burden of foodborne illnesses in humans [23]. Probably, that was why the CV673C and CV671C groups in this study had better protection against organ colonization and fecal shedding. However, Raut et al. [24] reported that several prophylactic measures should be implemented to reduce infection and egg contamination, including the use of effective biosecurity measures, stocking the farm with Salmonella-free replacement pullets, controlling rodent and insect vectors, and denying access to chick houses for wild birds and pets. Moreover, the prophylactic measures also comprised diligent cleaning and disinfection of chick houses before the new flocks were introduced. Eventually, the use of probiotics, prebiotics, synbiotics, immunization, and the refrigeration of shell eggs became important.

Consequently, it is possible to promote V673 and V671 for commercial use to control SEinfection in poultry. The control of SE from the fecal shedding by both combinations might bring on a significant impact, so long as vaccination is a strong prophylactic application against SE in laying chickens, especially when it is associated with the contaminated eggs [25]. This is because the eggs and egg-containing foods were the most frequently identified food vehicles [26]. However, in general, killed SE vaccines were able to decrease the presence of SE in the birds and in the eggs as well. Nevertheless, they must be associated with general hygiene and disinfection practices in poultry husbandry [27].

The macroscopic changes (gross lesions) and microscopic changes (histopathological lesions) observed in this study were consistent with the results of *Salmonella* detection, indicating that SE was not persistent in the tissues. Thus, gross lesions were absent in different tissues. The mild histopathological changes indicated that SE was eliminated from the tissue within a short time, and the bacteria were unable to multiply in the tissues. The efficacy of these combinations could be improved by double immunization and administration at a mature age.

In one previous study [28], it was found that a significant protection against SE infection and a reduction in the fecal shedding, invasion, and colonization of the microbe could be carried out with the combination of a killed vaccine with a live *S*. *Gallinarum* 9R vaccine. In other research conducted by Kang et al. [29], a subunit vaccine was successfully developed against *S. enterica* serovar Enteritidis by amplifying a gene (SseB) from the genomic DNA and then expressing the recombinant proteins (rHis-SseB and rGST-SseB) with the system of *Escherichia coli*. The recombinant proteins (rHis-SseB), in addition to a drug (simvastatin, a lipid-lowering medication), resulted in protection of 60% against the following pathogenic dose of SE and reduction of hepatic and splenic *Salmonella* colonization [29].

## Conclusion

The study demonstrated that all combinations of killed SEPTs used in the study could protect the chick against SE infection. However, V673 and V671 products are safer and more effective than other products in preventing and controlling SE infection in chicks.

## List of abbreviations

°C, degree Celsius; cfu, colony-forming unit; ELISA, enzyme-linked immunosorbent assay; gm, gram; ml, milliliter; MHC, major histocompatibility complex; SEPT, Salmonella Enteritidis phage type; SE, *Salmonella* Enteritidis; SEM, standard error of the mean; *p* < 0.05, 5% probability of observing the data (or more extreme results) if the null hypothesis were true; PT, phage type; pp, post-pathogenic; SE PT6A pa, *Salmonella* Enteritidis phage type 6A post-administration.
